# Predictive Properties of Plasma Amino Acid Profile for Cardiovascular Disease in Patients with Type 2 Diabetes

**DOI:** 10.1371/journal.pone.0101219

**Published:** 2014-06-27

**Authors:** Shinji Kume, Shin-ichi Araki, Nobukazu Ono, Atsuko Shinhara, Takahiko Muramatsu, Hisazumi Araki, Keiji Isshiki, Kazuki Nakamura, Hiroshi Miyano, Daisuke Koya, Masakazu Haneda, Satoshi Ugi, Hiromichi Kawai, Atsunori Kashiwagi, Takashi Uzu, Hiroshi Maegawa

**Affiliations:** 1 Department of Medicine, Shiga University of Medical Science, Otsu, Shiga, Japan; 2 Institute for Innovation, Ajinomoto Co., Inc., Kawasaki, Kanagawa, Japan; 3 Division of Diabetology & Endocrinology, Kanazawa Medical University, Kahoku-Gun, Ishikawa, Japan; 4 Division of Metabolism and Biosystemic Science, Department of Medicine, Asahikawa Medical University, Asahikawa, Hokkaido, Japan; Steno Diabetes Center, Denmark

## Abstract

Prevention of cardiovascular disease (CVD) is an important therapeutic object of diabetes care. This study assessed whether an index based on plasma free amino acid (PFAA) profiles could predict the onset of CVD in diabetic patients. The baseline concentrations of 31 PFAAs were measured with high-performance liquid chromatography-electrospray ionization-mass spectrometry in 385 Japanese patients with type 2 diabetes registered in 2001 for our prospective observational follow-up study. During 10 years of follow-up, 63 patients developed cardiovascular composite endpoints (myocardial infarction, angina pectoris, worsening of heart failure and stroke). Using the PFAA profiles and clinical information, an index (CVD-AI) consisting of six amino acids to predict the onset of any endpoints was retrospectively constructed. CVD-AI levels were significantly higher in patients who did than did not develop CVD. The area under the receiver-operator characteristic curve of CVD-AI (0.72 [95% confidence interval (CI): 0.64–0.79]) showed equal or slightly better discriminatory capacity than urinary albumin excretion rate (0.69 [95% CI: 0.62–0.77]) on predicting endpoints. A multivariate Cox proportional hazards regression analysis showed that the high level of CVD-AI was identified as an independent risk factor for CVD (adjusted hazard ratio: 2.86 [95% CI: 1.57–5.19]). This predictive effect of CVD-AI was observed even in patients with normoalbuminuria, as well as those with albuminuria. In conclusion, these results suggest that CVD-AI based on PFAA profiles is useful for identifying diabetic patients at risk for CVD regardless of the degree of albuminuria, or for improving the discriminative capability by combining it with albuminuria.

## Introduction

Cardiovascular disease (CVD) is a life-threatening complication in patients with diabetes. Since hyperglycemia, hypertension, and dyslipidemia are well recognized as conventional risk factors for CVD, early intervention against them is important to prevent the onset of CVD in this population [1]. Several clinical studies have indicated that the incidence of CVD in patients with type 2 diabetes could be reduced with intensive management for these risk factors [2], [3]. The development of biomarkers or an index to identify patients at high risk for CVD is also clinically important as it makes possible the initiation of adequate medication for patients at risk. Excessive urinary albumin excretion, called albuminuria, has been established as a reliable surrogate biomarker for CVD, because an increase or decrease in albuminuria has been reported to directly affect the incidence of CVD [3]–[5]. Thus, the prevention and reduction of albuminuria by intensive control of the above-mentioned conventional risk factors for CVD is considered an important therapeutic target in the care of patients with diabetes [2], [6], [7]. Despite these efforts, however, many patients still develop CVD, suggesting that only the evaluation of known risk factors is insufficient to distinguish between patients at high and low risk of CVD. It is therefore important that an additional predictive biomarker or index be found to identify those patients with diabetes who are at risk for CVD.

Recent studies have reported that alteration of plasma metabolomics profiles is significantly associated with certain disease conditions and can predict future development of diseases [8]–[11]. Among the numerous metabolites, plasma free amino acids (PFAAs) may be potent metabolites that have potential as excellent disease biomarkers because circulating free amino acids are involved in protein synthesis, organ networks, and as metabolic regulators of physiological states [12]. Recent technological advances have made possible the highly accurate analysis of PFAA levels using high-performance liquid chromatography-electrospray ionization-mass spectrometry (HPLC-ESI-MS) [13]. We have previously reported on the possibility of this technical approach being able to distinguish patients with lung cancer [14].

In the current study, we hypothesized that alterations in PFAA profiles may be early markers for identifying diabetic patients at risk for CVD. We measured PFAA profiles in plasma samples of patients with type 2 diabetes enrolled in our ongoing prospective observational follow-up study. We retrospectively investigated whether we could construct a diagnostic index based on these PFAA profiles, known as “AminoIndex™ (AI) technology” [12]–[14], and whether this index could predict the onset of CVD in patients with type 2 diabetes followed up for 10 years.

## Materials and Methods

### Ethics statement

The study protocol and informed consent procedure were approved by the Ethics Committee of Shiga University of Medical Science (Shiga, Japan) and Ajinomoto Co., Inc. (Kawasaki, Japan). This study was conducted according to the principles expressed in the Declaration of Helsinki. The raw data used in this study have not been deposited in a public database. This is in compliance with the agreement with the Ethics Committee.

### Subjects

This study was a retrospective analysis of samples obtained during our ongoing prospective observational study, the Shiga Prospective Observational Follow-up Study [15]. This prospective follow-up study was launched in 1996 to assess patient characteristics associated with the development and progression of diabetic complications, and to identify biomarkers and genetic factors that can be used in the early detection of diabetic patients at risk for these complications.

Diabetic patients who agreed to participate in this study and provided written informed consent were asked to provide a 24-h urine sample at baseline. Baseline blood samples were obtained after an overnight fast in tubes containing ethylenediaminetetraacetic acid. Plasma was prepared by centrifuging the blood samples at 3,000 rpm at 4°C for 15 min. If not analyzed immediately, plasma and urine samples were stored at −80°C. All participants underwent annual standardized clinical examinations and biochemical tests. Each patient's medical records were reviewed annually and the occurrence of CVD, cancer, and other diseases was confirmed.

To assess whether an amino acid-based index could better predict the onset of CVD (CVD-AI) over 10 years than albuminuria and other conventional risk factors, we analyzed samples from 420 Japanese patients with type 2 diabetes registered in this prospective trial in 2001. The PFAAs in the stored plasma samples of each eligible patient were measured. Six patients were excluded from the study because their PFAAs could not be accurately measured, and seven patients were excluded because their urine samples were not available. In addition, 22 patients with a previous history at baseline of cancer, collagen disease, CVD within the previous year, infectious disease, or non-diabetic kidney disease confirmed by a renal biopsy were excluded. Thus, the data from 385 patients were finally analyzed in this study.

### Definition of cardiovascular composite endpoints and clinical parameters

Cardiovascular composite endpoints were myocardial infarction, angina pectoris, worsening of congestive heart failure, and stroke [15]. Myocardial infarction was defined as a clinical presentation characterized by typical symptoms, electrocardiographic changes associated with an elevation of cardiac biomarkers, and angiographic evidence of coronary thrombosis. Angina pectoris was defined as the presence on imaging of lesions in patients with a history of typical chest pain or electrocardiographic changes, and invasive cardiovascular interventions. A worsening of congestive heart failure was defined as events requiring hospitalization for worsening typical symptoms of heart failure validated by echocardiography, not due to valvular heart disease or arrhythmia. Stroke, including ischemic stroke and cerebral hemorrhage, was defined as a persistent focal neurological symptom in which the onset was sudden and was not due to trauma or a tumor, and where the responsible lesion was detected on imaging modalities. If a patient died, his/her medical records were checked to identify the cause of death. If the cause of death was not clear, it was not considered related to CVD.

Based on the urinary albumin excretion rate (UAER) at baseline, patients were classified as having normoalbuminuria (UAER <20 µg/min, n = 265) or albuminuria (20 µg/min ≤ UAER, n = 120). Serum levels of creatinine were measured via an enzymatic method. Estimated glomerular filtration rate (eGFR) was calculated using the simplified equation proposed by the Japanese Society of Nephrology [16]: eGFR (ml/min/1.73 m^2^)  = 194× [age (years)]^−0.287^× [serum creatinine (mg/dl)] ^−1.094^×0.739 (for female patients). Hemoglobin A_1c_ (HbA1c) levels were those of the National Glycohemoglobin Standardization Program, according to the recommendations of the Japanese Diabetes Society [17]. Hypertension was defined as blood pressure (BP) ≥140/90 mmHg or current use of antihypertensive drugs. Brachial-ankle pulse wave velocity (baPWV) was measured by an automatic device (BP-203RPE; Colin, Komaki, Japan), with the higher of the right and the left values used in calculations.

### PFAAs and CVD-AI

Plasma samples were deproteinized using acetonitrile at a final concentration of 80%, and amino acid levels in plasma were measured by HPLC-ESI-MS/MS, followed by precolumn derivatization, as described [13], [14]. The concentrations of 31 amino acids were measured at the Institute for Innovation of the Ajinomoto Co., Inc. (Kawasaki, Japan).

Although the levels of PFAAs may differ significantly between cases and controls, the differences in individual amino acids are not always sufficiently discriminatory [14]. We therefore constructed a diagnostic index based on PFAA levels, known as “AminoIndex ™ technology” [12]–[14], to compress multidimensional information from PFAA profiles into a single dimension and to maximize the differences between cases and controls. The CVD-AI index was defined as the logarithmic odds ratio of CVD probability estimated by logistic regression models. Briefly, we generated all possible models with six or fewer variables. During this step, all possible combinations of variables were considered from a total of 31 amino acids [14]. Next, we calculated the area under the curve (AUC) for receiver-operator characteristic (ROC) curve analysis for all models with non-validation or leave-one-out cross validation (LOOCV). The model which produced the highest AUC for ROC curve analysis by LOOCV was selected as the final model, CDV-AI. Table S1 explains the top 10 models' performances using the AUC for ROC curve analysis.

### Statistical analysis

Clinical data are expressed as mean ± SD or median (interquartile range), as appropriate. Categorical variables were compared using χ^2^ tests, normally distributed continuous variables using unpaired Student's *t*-tests, and abnormally distributed continuous variables using the Mann–Whitney U test. In particular, differences in amino acid levels and the CVD-AI between the two groups were assessed by the Mann–Whitney U test. Spearman's rank correlation coefficient (ρ) was used to assess the correlation between each amino acid level and clinical variables. ROC curve analysis was performed to determine the capability and cut-off level of variables that distinguished between cases and controls. The 95% confidence interval (CI) of the AUC for ROC was also estimated. The unadjusted (crude) and adjusted hazard ratios (HR) for the occurrence of cardiovascular events were evaluated using a Cox proportional hazards regression model. Follow-up time was censored if any cardiovascular composite endpoint occurred or if the patient was unavailable for follow-up. To assess the risk factors for the cardiovascular composite endpoint, each variable listed in Table 1 and the CVD-AI were first evaluated using univariate analysis of the Cox proportional hazards regression model and then each estimate was adjusted for all variables showing statistical significance in the univariate model. To assess the combination effect of the CVD-AI and albuminuria, estimates were adjusted for the conventional risk factors of cardiovascular disease, including age, sex, HbA1c, total cholesterol, triglyceride, high density lipoprotein (HDL)-cholesterol, eGFR, body mass index (BMI) and hypertension. All analyses were performed using the SPSS software package (version 22; SPSS Inc., Chicago, IL, USA), with a two-sided *P* value of <0.05 considered statistically significant.

**Table 1:** Baseline characteristics of patients who did (cases) and did not (controls) experience cardiovascular events during follow-up.

Variables	Controls	Cases	*P* value
Number (n)	322	63	
Age (year)	60±12	68±7	<0.01
Gender (male/female, n)	156/166	35/28	0.30
Body mass index (kg/m^2^)	23.5±3.9	24.4±4.1	0.10
Hemoglobin A1c (%)	7.6±0.9	7.7±1.0	0.11
Total cholesterol (mg/dL)	206±30	207±29	0.70
Triglyceride (mg/dL)	92 (64–134)	104 (73–140)	0.14
HDL-cholesterol (mg/dL)	54 (46–65)	47 (41–55)	<0.01
Systolic blood pressure (mmHg)	135±19	143±20	<0.01
Diastolic blood pressure (mmHg)	76±11	75±10	0.49
Hypertension (%)	53.4	84.1	<0.01
Estimated GFR (ml/min/1.73m^2^)	82±23	67±21	<0.01
Urinary albumin excretion rate (µg/min)	8.4 (5.1–24.8)	27.4 (8.3–113.2)	<0.01
Albuminuria (%)	26.4	55.6	<0.01
baPWV (m/sec)	1786±522	1974±567	<0.01
Myocardial infarction (n)	-	11	-
Angina pectoris (n)	-	29	-
Congestive heart failure (n)	-	5	-
Stroke (n)	-	18	-

Data are expressed as mean ± SD for normally distributed continuous variables or median (interquartile range) for skewed continuous variables.

*Abbreviations:* GFR, glomerular filtration rate; HDL, high density lipoprotein; baPWV, brachial-ankle pulse wave velocity.

## Results

### Characteristics of subjects

During the 10-year follow-up period, 63 patients experienced cardiovascular endpoints, including 11 with myocardial infarction, 29 with angina pectoris, five with worsening of congestive heart failure, and 18 with stroke (Table 1). The clinical characteristics at baseline of these 63 patients with outcomes (cases) and the 322 without outcomes (controls) are presented in Table 1. Age, HDL-cholesterol, systolic BP, eGFR, UAER, and baPWV differed significantly in these two groups.

### PFAA profiles related to cardiovascular composite endpoints

The mean levels of each amino acid in the case and control groups are shown in Table 2. Among the 31 amino acids tested, three (β-amino-iso-butyric acid, 3-methylhistidine, and citrulline) were significantly higher and one (tryptophan) was significantly lower in cases than in controls. Some amino acid levels showed statistically significant correlations with some clinical variables related to risk factors of cardiovascular disease (Table S2).

**Table 2:** Absolute levels of 31 plasma amino acids in patients who did (cases) and did not (controls) experience cardiovascular events during follow-up.

Amino Acids (µmol/l)	HMDB ID	Control (n = 328)	Case (n = 63)	*P* value
3-methylhistidine (3MeHis)	HMDB01861	2.3±1.6	3.5±2.8	0.001
Citrulline (Cit)	HMDB00904	38.0±13.2	43.1±15.1	0.016
Tryptophan (Trp)	HMDB00929	60.5±13.0	55.3±13.0	0.016
β-amino-iso-butyric acid (β-AIBA)	Not available	1.6±2.0	2.2±2.3	0.033
Cystine (Cys)	HMDB00192	55.8±13.7	60.0±15.5	0.048
Glutamic acid (Glu)	HMDB00148	33.2±14.7	37.1±16.2	0.078
α-amino adipic acid (α-AAA)	HMDB00510	0.3±0.6	0.4±0.7	0.105
Threonine (Thr)	HMDB00167	130.0±31.2	122.2±32.2	0.118
Methionine (Met)	HMDB00696	27.5±5.8	26.5±5.7	0.153
Serine (Ser)	HMDB00187	116.8±22.5	112.3±21.3	0.160
Histidine (His)	HMDB00177	81.3±11.8	79.1±10.8	0.205
Ethanolamine (EtOHNH2)	HMDB00149	6.9±1.6	6.6±1.5	0.242
Prorine (Pro)	HMDB00162	150.6±41.0	157.3±42.6	0.298
Taurine (Tau)	HMDB00251	61.4±15.1	62.3±13.0	0.307
Arginine (Arg)	HMDB00517	102.4±24.4	105.9±25.9	0.374
Hydroxyproline (HyPro)	HMDB00725	12.3±6.2	12.9±6.2	0.385
Aspartic acid (Asp)	HMDB00191	2.3±1.1	2.4±1.0	0.401
Asparagine (Asn)	HMDB00168	51.8±10.	51.0±10.	0.402
Phenylalanine (Phe)	HMDB00159	67.2±11.1	66.8±12.6	0.519
Ornithine (Orn)	HMDB00214	65.7±16.2	69.2±21.3	0.564
Tyrosine (Tyr)	HMDB00158	78.3±19.2	76.2±18.4	0.599
α-amino-n-butyric acid (α-ABA)	Not available	22.4±6.7	22.1±7.5	0.722
Valine (Val)	HMDB00883	250.3±45.8	245.5±43.5	0.741
Isoleucine (Ile)	HMDB00172	75.9±17.1	75.8±15.9	0.838
Glycine (Gly)	HMDB00123	227.2±54.9	228.5±53.8	0.851
Sarcosine (Sar)	HMDB00271	2.1±1.0	2.1±1.1	0.876
Glutamine (Gln)	HMDB00641	622.7±120.4	631.4±154.8	0.876
Leucine (Leu)	HMDB00687	137.0±27.2	135.2±26.3	0.926
1-methylhistidine (1MeHis)	HMDB00001	4.2±5.4	4.5±6.2	0.953
Alanine (Ala)	HMDB00161	428.7±94.6	427.5±93.1	0.954
Lysine (Lys)	HMDB00182	197.9±37.1	200.0±43.1	0.974

*Abbreviations:* HMDB ID: Human Metabolome Database ID.

### Predictive effect of CVD-AI

We next assessed whether the onset of the cardiovascular composite endpoint could be distinguished by the multivariate analysis referred to as AI technology. Using this technology, the optimal CVD-AI was constructed from the data set of PFAAs: CVD-AI  =  (−0.1452) + (−0.2230) × (ethanolamine) + (−0.04637) × (hydroxyproline) + (0.01303) × (glutamic acid) + (0.3524) × (3-methylhistidine) + (0.01250) × (tyrosine) + (−0.03093) × (tryptophan).

The mean value of the CVD-AI was significantly higher in cases than in controls (−1.28±0.94 *vs.* −1.90±0.69, *P*<0.001). The CVD-AI value was positively correlated with age (ρ = 0.23, *P*<0.01), BMI (ρ = 0.15, *P*<0.01), triglyceride (ρ = 0.18, *P*<0.01), systolic BP (ρ = 0.18, *P*<0.01), baPWV (ρ = 0.22, *P*<0.001) and UAER (ρ = 0.30, *P*<0.001), and inversely correlated with HDL-cholesterol (ρ = −0.21, *P*<0.001) and eGFR (ρ = −0.39, *P*<0.001), although it was not correlated with HbA1c level (ρ = 0.08, *P* = 0.12). Furthermore, the CVD-AI values in patients with antihypertensive agents were higher than those without (−1.54±0.88 *vs.* −1.97±0.63, *P*<0.001), whereas the CVD-AI values were not different among the three patient subgroups stratified by antidiabetic medication (diet only, oral agents and insulin therapy).

Compared with the AUC for ROC curve analysis, the CVD-AI showed better discriminatory ability (0.72 [95% CI: 0.64–0.79]) than did the level of each amino acid (Table 3). Even when validated by LOOCV analysis, the AUC of the CVD-AI ROC was 0.68. ROC curve analysis showed that the CVD-AI cut-off level for this outcome was −1.662. In Cox proportional hazards regression analysis, patients with the CVD-AI above the cut-off level showed a significantly higher unadjusted HR of 4.62 (95% CI: 2.65–8.04) for the cardiovascular composite endpoint, as did age, systolic BP, hypertension, HDL, UAER, eGFR, and baPWV (Table 4). Even when adjusted for these variables shown to be statistically significant in the univariate model, the CVD-AI, as well as age and UAER, was identified as an independent risk for this outcome (adjusted HR: 2.86, [95% CI: 1.57–5.19], Table 4).

**Table 3:** Areas under the receiver-operating characteristic curves distinguishing patients who did (cases) and did not (controls) experience cardiovascular events during follow-up.

Parameters	AUC (± SE)	(95% CI)	*P* value
β-AIBA (µmol/l)	0.59±0.04	(0.50–0.66)	0.002
3MeHis (µmol/l)	0.62±0.04	(0.55–0.71)	0.04
Cit (µmol/l)	0.59±0.04	(0.52–0.07)	0.04
Trp (µmol/l)	0.59±0.04	(0.52–0.67)	0.02
CVD-AI	0.72±0.04	(0.64–0.79)	<0.0001
UAER	0.69±0.04	(0.62–0.77)	<0.0001

*Abbreviations:* AUC, area under the receiver-operator characteristic curve; β-AIBA, β-amino-iso-butyric acid; 3MeHis, 3-methylhistidine; Cit, citrulline; Trp, tryptophan; Cys, cystine; CVD-AI, cardiovascular disease-amino acid based index; UAER, urinary albumin excretion rate.

**Table 4:** Hazard ratios for the cardiovascular composite endpoint.

	Univariate model		Multivariate model a	
	Hazard ratio (95% CI)	*P* value	Adjusted Hazard ratio (95% CI)	*P* value
Age (year)	1.08 (1.05–1.11)	< 0.001	1.07 (1.04–1.11)	<0.001
Systolic BP (mmHg)	1.02 (1.01–1.04)	0.001	0.99 (0.99–1.02)	0.85
Hypertension (yes)	4.08 (2.07–8.05)	<0.001	2.06 (0.95–4.46)	0.07
Log HDL-cholesterol (mg/dl)	0.02 (0.01–0.26)	0.002	0.16 (0.01–2.00)	0.16
Log UAER (µg/min)	2.14 (1.60–2.92)	<0.001	1.56 (1.04–2.35)	0.03
eGFR (ml/min/1.73 m^2^)	0.97 (0.96–0.99)	<0.001	1.00 (0.99–1.02)	0.63
baPWV (m/sec)	1.00 (1.00–1.01)	0.008	1.00 (0.99–1.00)	0.42
CVD-AI	4.62 (2.65–8.04)	<0.001	2.86 (1.57–5.19)	0.001

The variables listed in Table 1 and CVD-AI were firstly assessed in the univariate analysis of the Cox proportional hazards regression model. Only variables shown to be statistically significant in the univariate model are shown in this table.

a Each estimate was adjusted for all variables shown in this table.

*Abbreviations:* BP, blood pressure; CI, confidence interval, CVD-AI, cardiovascular disease-amino acid based index; HDL, high density lipoprotein; UAER, urinary albumin excretion rate; eGFR, estimated glomerular filtration rate; baPWV, brachial-ankle pulse wave velocity.

Next, we separately estimated the risk of the CVD-AI for two conditions: coronary vascular events (myocardial infarction and angina pectoris, n = 40) and cerebrovascular events (stroke, n = 18). Unadjusted HR for coronary vascular events was 5.51 (95% CI: 2.85–10.64). Adjusted for variables listed in Table 4, the risk of the CVD-AI for coronary vascular events did not change (adjusted HR: 3.35 [95% CI: 1.64–6.83]). In contrast, the unadjusted and adjusted HR for stroke were 2.61 (95% CI: 0.99–6.85) and 1.51 (95% CI: 0.52–4.37), respectively.

### Combination effect of UAER and CVD-AI

In this study, UAER has also been identified as an independent risk for cardiovascular outcome, as in previous reports, and the AUC for ROC curve analysis of UAER (0.69 [95% CI: 0.62–0.77]) was almost equally to that of the CVD-AI (0.72 [95% CI: 0.64–0.79]). We thus finally analyzed the combination effect of UAER and CVD-AI in predicting cardiovascular composite endpoints. For this purpose, patients were divided into four subgroups: those with normoalbuminuria and above or below the cut-off level of CVD-AI and those with albuminuria and above or below the cut-off level of CVD-AI (Table 5). In patients with a CVD-AI above the cut-off level, both those with normoalbuminuria (unadjusted HR: 3.24 [95% CI: 1.54–6.82]) and albuminuria (unadjusted HR: 8.25 [95% CI: 4.28–15.9]) were at significantly higher risk for the onset of cardiovascular composite endpoints. Even after adjustment for the conventional risk factors of cardiovascular disease, both groups remained at risk (Table 5). In contrast, patients with a CVD-AI below the cut-off level, even those with albuminuria (HR: 1.48 [95% CI: 0.55–3.99]), were not at significant risk for this outcome.

**Table 5:** Crude and multivariate-adjusted hazard ratios for the cardiovascular composite endpoint in patient subgroups stratified according to urinary albumin excretion rate and the CVD-AI.

Subgroup category	Total (n)	Case (n)	Crude Hazard ratio (95% CI)	*P* value	Adjusted Hazard ratio a (95% CI)	*P* value
UAER <20 µg/min + Low CVD-AI	192	13	1.00 (reference)		1.00 (reference)	
UAER <20 µg/min + High CVD-AI	72	15	3.24 (1.54–6.82)	0.002	2.61 (1.23–5.54)	0.012
UAER ≥20 µg/min + Low CVD-AI	54	6	1.76 (0.67–4.63)	0.25	1.48 (0.55–3.99)	0.44
UAER ≥20 µg/min + High CVD-AI	63	29	8.25 (4.28–15.9)	<0.001	4.52 (2.09–9.80)	<0.001

Subjects were categorized as being above or below a UAER of 20 µg/min and above or below the CVD-AI cut-off value of −1.662. Crude (unadjusted) and adjusted hazard ratios were calculated using Cox proportional hazards regression models.

a Estimates were adjusted for the conventional risk factors of cardiovascular disease, including age, sex, HbA1c, total cholesterol, triglyceride, high density lipoprotein cholesterol, estimated glomerular filtration rate, body mass index and hypertension.

*Abbreviations:* CVD-AI, cardiovascular disease-amino acid based index; UAER, urinary albumin excretion rate.

**Figure 1:**
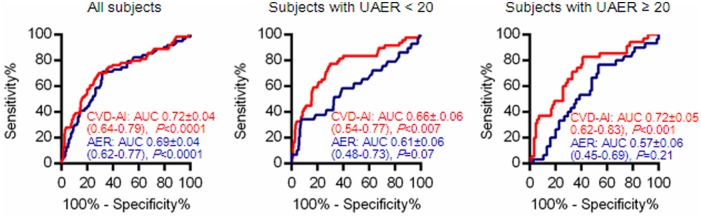
Results of area under the curve of receiver-operator characteristics curve analysis for both CVD-AI and urinary album excretion rate to distinguish cases from controls in all subjects and those with/without albuminuria.

We found that the CVD-AI could distinguish cases from controls even when patients with normoalbuminuria (AUC: 0.66, 95% CI: 0.54–0.77, *P* = 0.007) and those with albuminuria (AUC: 0.72, 95% CI: 0.62–0.83, *P*<0.001) were separately analyzed (Figure 1). In contrast, UAER was unable to distinguish cases from controls, both in patients with normoalbuminuria (AUC: 0.61, 95% CI: 0.48–0.73, *P* = 0.07) and those with albuminuria (AUC: 0.59, 95% CI: 0.46–0.69, *P* = 0.21).

## Discussion

Identification of a reliable surrogate marker or index for predicting the onset of CVD is essential in the care of patients with diabetes. Using high-throughput PFAA profiling and the data of our ongoing prospective observational follow-up study we constructed the diagnostic index, the CVD-AI, to predict the onset of CVD in patients with type 2 diabetes. Interestingly, this predictive effect was independent of the levels of albuminuria and the conventional risk factors of CVD, indicating that altered PFAA profiles were able to effectively identify high risk patients, even those without albuminuria. These findings suggest that the PFAA profile is a clinically useful index for improving the discriminative capability for coronary artery disease in diabetic patients in addition to conventional risk factors and better risk stratification even among those with normoalbuminuria, who are at relatively low risk for CVD.

Alterations in the composition of PFAAs have been reported to reflect the pathological status or preconditions in numerous diseases including CVD, suggesting that these alterations may be involved in disease development processes [18]–[21]. Several clinical studies using this new technology have reported on the association between the altered composition of PPFAs and the predictive effect for CVD. Shah *et al.* demonstrated that plasma metabolomic profiles, including several amino acids, have been found to predict cardiovascular events and improve risk discrimination beyond the degree possible using readily available clinical characteristics [20], [21]. Magnusson *et al.* also reported that an amino acid index consisting of branched-chain and aromatic amino acids was found to strongly predict the development of CVD during 12 years of follow-up [19]. As with these previous reports, branched-chain amino acids and aromatic amino acids in the current study were found to correlate with obesity- and dyslipidemia-related risks for CVD. However, the predictive power of each amino acid for CVD was relatively weak, although some amino acids showed significantly different plasma levels between cases and controls. The CVD-AI based on the PFAA profiles, called “AminoIndex™ technology” [11]–[13], improved the predictive effect for CVD in comparison to individual PFAAs. These results suggest that the CVD-AI is a more sensitive and effective predictive index than the conventional risk factors to identify patients at risk for CVD, although we need to validate the predictive effect of this CVD-AI.

The ability to identify patients at high risk of CVD before its onset is particularly important in diabetes care, because CVD can greatly affect mortality and quality of life in patients with diabetes. Albuminuria is a strong predictor for CVD, making the prevention of increased albuminuria and the reduction of albuminuria a therapeutic target for the prevention of CVD [2]–[7]. Although albuminuria was one of the risk factors for CVD in our population, as well as in previous reports, the CVD-AI showed almost equal or slightly better discriminatory capability than UAER in ROC curve analysis. In addition, the CVD-AI was identified as an independent risk factor for the onset of CVD even after adjusting the conventional risk factors including albuminuria in the Cox proportional hazards regression model. Interestingly, this predictive effect was observed even in patients with normoalbuminuria as well as those with albuminuria. Thus, PFAA profiles may be clinically useful as a novel index for identifying diabetic patients at high risk for CVD regardless of the degree of albuminuria or improving the discriminative capability by combining it with albuminuria.

It remains unclear whether the association between altered PFAA profiles and CVD onset represents a cause-effect relationship. Metabolic profiles have been reported to be highly heritable in families with early-onset CVD [22]. Thus, the susceptibility of diabetic patients to the onset of CVD may be due in part to genetically determined metabolic components. In this study, the CVD-AI significantly correlated with cardiovascular risk factors, particularly dyslipidemia, renal function and hypertension, whereas it did not correlate with HbA1c. This may mean that the CVD-AI reflects the influence of atherosclerosis rather than glycemic control. Also, amino acids are reported to directly contribute to insulin resistance by disrupting insulin signaling [23]. Because insulin resistance promotes the development of atherosclerosis, the altered PFAA profiles associated with insulin resistance may be indirectly associated with the onset of CVD. Unfortunately, we could not investigate the association between the CVD-AI and insulin resistance in this study. Further studies are needed to clarify whether the CVD-AI is a specific index for patients with diabetes mellitus.

This study had several limitations. This study was designed as a retrospective analysis of samples and data obtained during our prospective observational follow-up study, not as an interventional study. Thus, treatment protocols including dietary regimens were not controlled, and the influence of cofounders during the observational period was not analyzed. The time-dependent changes in PFAAs during follow-up periods were also not assessed. Therefore, it remains unclear as to whether the correction of these altered PFAA profiles represents a new therapeutic target to prevent CVD in patients with diabetes. Furthermore, we need to validate the CVD-AI using the PFAA profiles identified in this study, and further prospective studies are required to confirm whether our CVD-AI is most suitable for predicting the onset of CVD and to determine whether correcting the altered PFAA profiles can improve prognosis in patients with diabetes mellitus.

## Conclusions

This study has demonstrated that altered PFAA profiles can predict the onset of CVD in patients with type 2 diabetes over a 10-year follow-up period. These alterations predicted the onset of CVD regardless of the degree of albuminuria and other conventional risk factors for CVD. Further prospective studies are required to validate the clinical utility of these PFAA measurements and to construct an optimal CVD-AI that can be used to identify diabetic patients at high risk for CVD in clinical practice.

## Acknowledgments

We are grateful to all members of the Maegawa Laboratory for their scientific input and contributions to the Shiga Prospective Observational Follow-up Study. We also thank Yumiko Omura and Keiko Kondo (Shiga University of Medical Science) for their excellent assistance.

## Supporting Information

Table S1 **The top ten models performance using ROC of AUC.**
(PPTX)

Table S2 **Correlation between plasma level of each amino acid and conventional cardiovascular risk.**
(DOCX)
