# Supplementary material for: Predictive Properties of Plasma Amino Acid Profile for Cardiovascular Disease in Patients with Type 2 Diabetes
Source: PLoS One. 2014 Jun 27;9(6):e101219. doi: 10.1371/journal.pone.0101219 (PMC4074128; doi:10.1371/journal.pone.0101219)
Supplement: Table S1 — The top ten models performance using ROC of AUC. (PPTX) [file pone.0101219.s001.pptx]

## Slide 1
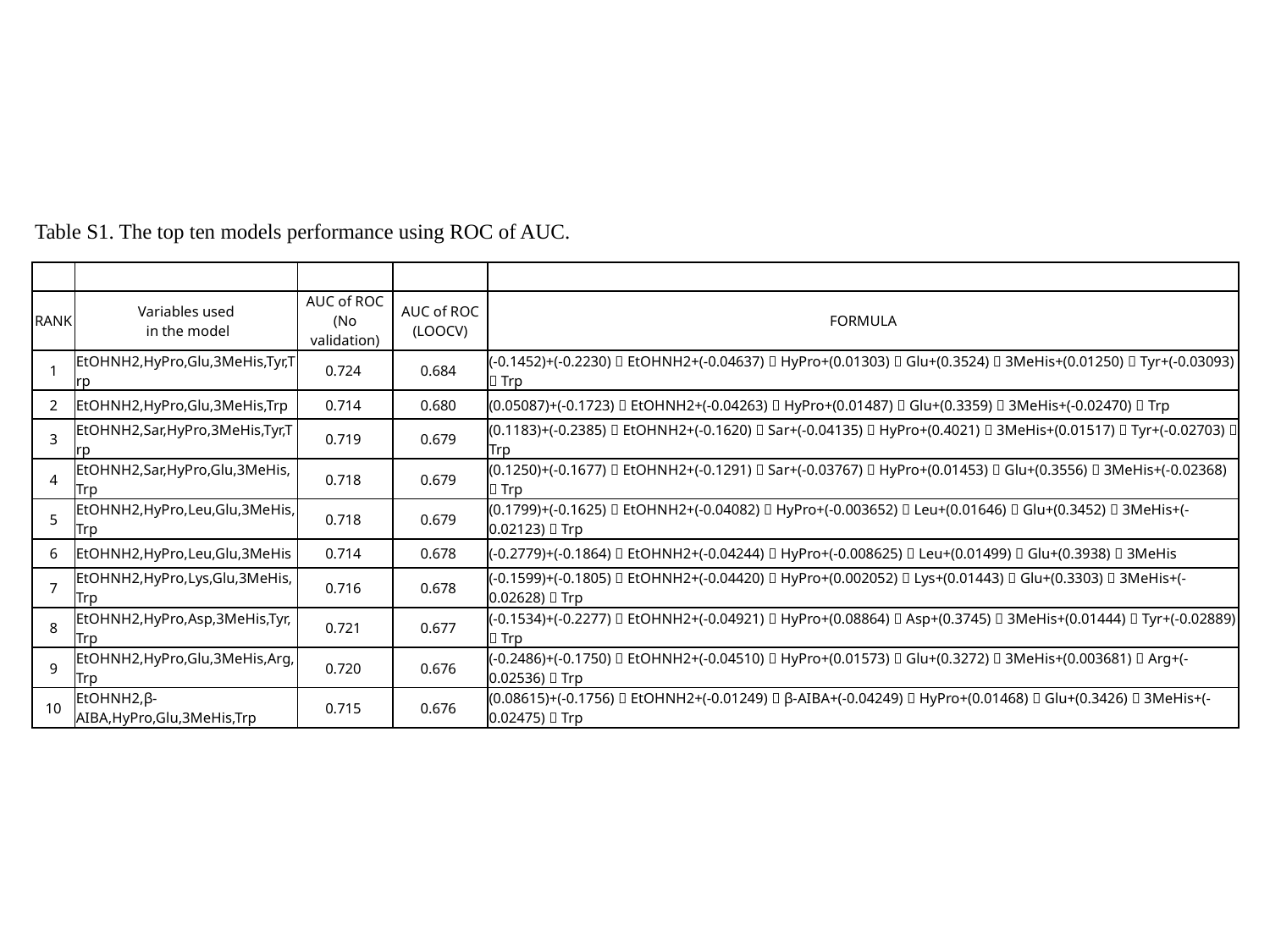

Table S1. The top ten models performance using ROC of AUC.
| | | | | |
| --- | --- | --- | --- | --- |
| RANK | Variables used in the model | AUC of ROC (No validation) | AUC of ROC (LOOCV) | FORMULA |
| 1 | EtOHNH2,HyPro,Glu,3MeHis,Tyr,Trp | 0.724 | 0.684 | (-0.1452)+(-0.2230)ｘEtOHNH2+(-0.04637)ｘHyPro+(0.01303)ｘGlu+(0.3524)ｘ3MeHis+(0.01250)ｘTyr+(-0.03093)ｘTrp |
| 2 | EtOHNH2,HyPro,Glu,3MeHis,Trp | 0.714 | 0.680 | (0.05087)+(-0.1723)ｘEtOHNH2+(-0.04263)ｘHyPro+(0.01487)ｘGlu+(0.3359)ｘ3MeHis+(-0.02470)ｘTrp |
| 3 | EtOHNH2,Sar,HyPro,3MeHis,Tyr,Trp | 0.719 | 0.679 | (0.1183)+(-0.2385)ｘEtOHNH2+(-0.1620)ｘSar+(-0.04135)ｘHyPro+(0.4021)ｘ3MeHis+(0.01517)ｘTyr+(-0.02703)ｘTrp |
| 4 | EtOHNH2,Sar,HyPro,Glu,3MeHis,Trp | 0.718 | 0.679 | (0.1250)+(-0.1677)ｘEtOHNH2+(-0.1291)ｘSar+(-0.03767)ｘHyPro+(0.01453)ｘGlu+(0.3556)ｘ3MeHis+(-0.02368)ｘTrp |
| 5 | EtOHNH2,HyPro,Leu,Glu,3MeHis,Trp | 0.718 | 0.679 | (0.1799)+(-0.1625)ｘEtOHNH2+(-0.04082)ｘHyPro+(-0.003652)ｘLeu+(0.01646)ｘGlu+(0.3452)ｘ3MeHis+(-0.02123)ｘTrp |
| 6 | EtOHNH2,HyPro,Leu,Glu,3MeHis | 0.714 | 0.678 | (-0.2779)+(-0.1864)ｘEtOHNH2+(-0.04244)ｘHyPro+(-0.008625)ｘLeu+(0.01499)ｘGlu+(0.3938)ｘ3MeHis |
| 7 | EtOHNH2,HyPro,Lys,Glu,3MeHis,Trp | 0.716 | 0.678 | (-0.1599)+(-0.1805)ｘEtOHNH2+(-0.04420)ｘHyPro+(0.002052)ｘLys+(0.01443)ｘGlu+(0.3303)ｘ3MeHis+(-0.02628)ｘTrp |
| 8 | EtOHNH2,HyPro,Asp,3MeHis,Tyr,Trp | 0.721 | 0.677 | (-0.1534)+(-0.2277)ｘEtOHNH2+(-0.04921)ｘHyPro+(0.08864)ｘAsp+(0.3745)ｘ3MeHis+(0.01444)ｘTyr+(-0.02889)ｘTrp |
| 9 | EtOHNH2,HyPro,Glu,3MeHis,Arg,Trp | 0.720 | 0.676 | (-0.2486)+(-0.1750)ｘEtOHNH2+(-0.04510)ｘHyPro+(0.01573)ｘGlu+(0.3272)ｘ3MeHis+(0.003681)ｘArg+(-0.02536)ｘTrp |
| 10 | EtOHNH2,β-AIBA,HyPro,Glu,3MeHis,Trp | 0.715 | 0.676 | (0.08615)+(-0.1756)ｘEtOHNH2+(-0.01249)ｘβ-AIBA+(-0.04249)ｘHyPro+(0.01468)ｘGlu+(0.3426)ｘ3MeHis+(-0.02475)ｘTrp |
