# Supplementary material for: Predictive Properties of Plasma Amino Acid Profile for Cardiovascular Disease in Patients with Type 2 Diabetes
Source: PLoS One. 2014 Jun 27;9(6):e101219. doi: 10.1371/journal.pone.0101219 (PMC4074128; doi:10.1371/journal.pone.0101219)
Supplement: Table S2 — Correlation between plasma level of each amino acid and conventional cardiovascular risk. (DOCX) [file pone.0101219.s002.docx]

Table S2. Correlation between plasma level of each amino acid and conventional cardiovascular risk.

| Amino Acids (μmol/L) | Age | HbA1c  (NGSP) | BMI | T-Cho | Log TG | LogHDL-Cho | UAER | eGFR | sysBP |
| --- | --- | --- | --- | --- | --- | --- | --- | --- | --- |
| 3-methylhistidine (3MeHis) | 0.089 | 0.028 | 0.173** | -0.017 | 0.164** | -0.172** | 0.338** | -0.504** | 0.135** |
| Citrulline (Cit) | 0.198** | 0.061 | -0.130* | -0.065 | -0.010 | 0.008 | 0.147 | -0.422** | -0.138** |
| Tryptophan (Trp) | -0.134** | -0.024 | 0.085 | -0.024 | 0.224** | -0.073 | -0.058 | 0.085 | 0.004 |
| β-amino-iso-butyric acid (β-AIBA) | 0.159** | -0.089 | -0.045 | -0.042 | 0.020 | -0.124* | 0.163** | -0.209** | 0.115* |
| Cystine (Cys) | 0.221** | 0.041 | 0.179** | -0.013 | 0.195** | -0.225** | 0.288** | -0.344** | 0.203** |
| Glutamic acid (Glu) | 0.068 | 0.142** | 0.336** | 0.070 | 0.447** | -0.304** | 0.188** | -0.113* | 0.163** |
| α-amino adipic acid (α-AAA) | -0.064 | 0.145** | 0.077 | -0.027 | 0.103* | -0.157** | 0.157** | -0.225** | 0.036 |
| Threonine (Thr) | -0.139** | -0.044 | 0.073 | -0.075 | -0.054 | -0.015 | 0.045 | -0.041 | 0.038 |
| Methionine (Met) | -0.098 | -0.022 | 0.088 | -0.141** | 0.030 | -0.024 | 0.0054 | -0.068 | 0.060 |
| Serine (Ser) | -0.082 | -0.062 | -0.139** | -0.064 | -0.277** | 0.083 | -0.155** | 0.238** | -0.075 |
| Histidine (His) | -0.112* | -0.130** | 0.153** | -0.105* | 0.069 | -0.029 | 0.083 | -0.086 | 0.025 |
| Ethanolamine (EtOHNH2) | -0.119* | 0.071 | 0.224** | 0.29 | 0.037 | 0.000 | 0.023 | -0.38 | 0.055 |
| Prorine (Pro) | -0.166** | -0.010 | 0.155** | -0.138** | 0.185** | -0.233** | 0.207** | -0.163** | 0.067 |
| Taurine (Tau) | 0.119* | 0.045 | 0.019 | 0.099 | 0.073 | -0.005 | 0.017 | -0.198** | 0.068 |
| Arginine (Arg) | 0.100* | -0.004 | -0.134** | -0.032 | -0.057 | -0.001 | 0.041 | -0.168** | 0.080 |
| Hydroxyproline (HyPro) | -0.007 | 0.017 | 0.075 | -0.089 | 0.040 | -0.096 | 0.204** | -0.265** | 0.046 |
| Aspartic acid (Asp) | 0.068 | 0.019 | 0.184** | 0.102* | 0.165** | -0.061 | -0.23 | -0.015 | 0.073 |
| Asparagine (Asn) | -0.121* | 0.034 | -0.123 | -0.143** | -0.060 | -0.048 | 0.043 | -0.035 | -0.053 |
| Phenylalanine (Phe) | 0.049 | -0.010 | 0.180** | -0.111* | 0.102* | -0.102* | 0.159** | -0.202** | 0.121* |
| Ornithine (Orn) | 0.135** | -0.013 | 0.026 | -0.092 | 0.006 | -0.128* | 0.079 | -0.173** | 0.091 |
| Tyrosine (Tyr) | 0.032 | 0.020 | 0.253** | -0.120* | 0.188** | -0.149** | -0.021 | -0.059 | 0.076 |
| α-amino-n-butyric acid (α-ABA) | -0.185** | 0.090 | 0.114* | 0.65 | -0.054 | 0.062 | 0.144** | 0.091 | 0.005 |
| Valine (Val) | -0.123 | 0.047 | 0.245** | -0.012 | 0.304** | -0.255** | 0.105* | -0.048 | 0.083 |
| Isoleucine (Ile) | -0.126* | 0.113* | 0.165** | -0.102* | 0.311** | -0.264** | 0.172** | -0.118* | 0.084 |
| Glycine (Gly) | -0.68 | 0.031 | -0.017 | -0.071 | 0.043 | -0.068 | -0.046 | -0.123* | 0.044 |
| Sarcosine (Sar) | -0.035 | 0.031 | -0.017 | -0.071 | 0.043 | -0.068 | 0.189** | -0.152* | 0.132** |
| Glutamine (Gln) | 0.191** | -0.101* | -0.078 | -0.075 | -0.075 | -0.068 | -0.036 | -0.152** | 0.127* |
| Leucine (Leu) | -0.198** | 0.110* | 0.190** | -0.094 | 0.258** | -0.216** | 0.158** | -0.032 | 0.037 |
| 1-methylhistidine (1MeHis) | 0.072 | -0.007 | 0.004 | -0.038 | 0.060 | -0.016 | 0.163** | -0.236** | 0.135** |
| Alanine (Ala) | -0.089 | 0.103* | 0.326** | 0.031 | 0.369** | -0.221** | 0.157** | -0.119* | 0.123* |
| Lysine (Lys) | 0.018 | -0.009 | 0.085 | -0.060 | 0.060 | -0.141** | 0.104* | -0.041 | -0.009 |

Data are expressed as Spearman's rank correlation coefficient (ρ) between the plasma levels of each amino acid and each clinical variable. *P < 0.05, **P < 0.01.

Abbreviation: HbA1c, hemoglobin A1c; BMI, body mass index; T-Cho, total cholesterol; TG; triglyceride; HDL-C, high density lipoprotein cholesterol; UAER, urinary albumin excretion rate; eGFR, estimated glomerular filtration rate; sysBP, systolic blood pressure
